# Sonic hedgehog promotes generation and maintenance of human forebrain Olig2 progenitors

**DOI:** 10.3389/fncel.2013.00254

**Published:** 2013-12-13

**Authors:** J. Alberto Ortega, Nevena V. Radonjić, Nada Zecevic

**Affiliations:** ^1^Department of Neuroscience, University of Connecticut Health CenterFarmington, CT, USA; ^2^Institute of Medical and Clinical Biochemistry, School of Medicine, University of BelgradeBelgrade, Serbia

**Keywords:** glia, cortical development, cellular fate, myelination, transcription factors

## Abstract

Function of oligodendrocytes (OLs), myelin forming cells in the CNS, is disrupted in demyelinating diseases such as periventricular leukomalacia or multiple sclerosis. It is, thus, important to better understand factors that can affect generation or differentiation of human OLs. In rodents, Sonic hedgehog (Shh) is influencing expression of Olig2, a helix-loop-helix transcription factor required for development of OLs. In humans, Olig2 is present in cortical progenitors at midgestation, however the role of Shh in the specification of human OLs, including Olig2 positive (Olig2^+^) progenitors, is not fully understood. Here we studied *in vitro* effects of Shh signaling on proliferation and specification of human cortical Olig2^+^ progenitors at midgestation. First, we established that the spatial pattern of Olig2 expression in the human developing CNS, described on cryosections, was preserved in mixed and enriched radial glia cell (RGC) cultures. Next, we demonstrated that *in vitro* treatment with Shh induced an increase in the number of Olig2^+^ progenitors. Shh treatment increased the density of early oligodendrocyte progenitors (OPCs) at the expense of RGC, while the number of late OPCs, did not change. However, inhibition of endogenous Shh with cyclopamine did not reduce the density of Olig2^+^ cells, implying the presence of an additional Shh-independent mechanism for OLs generation *in vitro*. These results suggest that the primary role of Shh signaling in the human dorsal oligodendrogenesis is the expansion and specification of multipotent radial glia progenitors into Olig2^+^ early OPCs. These results obtained *in vitro* are relevant to understand primary myelination during CNS development, as well as remyelination in demyelinating diseases.

## Introduction

The origin and differentiation of oligodendrocytes (OLs) have been extensively studied in animal models, and are especially well documented in rodents, mainly due to advances in genetic mapping of cell lineages (He et al., 2001; Marshall and Goldman, 2002; Fogarty et al., 2005; Kessaris et al., 2006). In the rodent telencephalon, the majority of embryonic OLs are generated in the ventral forebrain (ganglionic eminence, GE), but a subpopulation of OLs is also generated postnatally in the cortical subventricular zone (SVZ) (Levison and Goldman, 1993; Gorski et al., 2002; Kessaris et al., 2006; Richardson et al., 2006; Kessaris et al., 2008). Notably, it has been suggested that only dorsally generated OLs remain in the adult mouse brain (Kessaris et al., 2006). In the human developing brain, OLs differentiation proceeds through similar stages described in rodents (Rivkin et al., 1995; Back et al., 2001; Ulfig et al., 2002; Wilson et al., 2003; Jakovcevski and Zecevic, 2005; Jakovcevski et al., 2009). At midgestation, human oligodendrocyte progenitor cells (OPCs) originate both in the ventral and dorsal telencephalon (Rakic and Zecevic, 2003; Jakovcevski et al., 2009). Cells generated from these two sources could potentially differ in vulnerability or response to environmental factors. This might be clinically important in various diseases, from the ones that present with demyelination, such as multiple sclerosis (MS) or hereditary leukodistrophy, to complex neurological disorders such as Alzheimer's disease and schizophrenia (Goldman et al., 2012).

In rodents, radial glial cells (RGCs) and several intermediate precursor cells, presumably generated from RGCs, give rise to OLs in the developing CNS (Raff et al., 1998; Rao et al., 1998; Fogarty et al., 2005; Casper and Mccarthy, 2006; Kriegstein and Alvarez-Buylla, 2009). Along oligodendroglial lineage, cells change their morphologies and expression pattern of OLs- specific proteins. Early OPCs are bipolar migratory cells that express chondroitin-sulfate proteoglycan (NG2) and platelet-derived growth factor receptor alpha (PDGFRα). They differentiate into late OPCs which can be labeled with anti-O4 antibody, and finally into premyelinating and myelinating OLs recognized by anti-O1 antibody and antibodies to myelin basic protein (MBP) and proteolipid protein (PLP), respectively; these cells do not proliferate or migrate (Pfeiffer et al., 1993; De Castro and Bribián, 2005; Meijer et al., 2012).

In addition to similar sequential expression of immunohistochemical markers during the progression of OLs lineage in humans, we have reported that *in vitro* human fetal RGCs can generate OLs and that this process is enhanced by Sonic hedgehog (Shh) (Jakovcevski et al., 2009; Mo and Zecevic, 2009). Shh is an essential morphogen critical for normal development of the brain, especially for its ventral patterning (Gritli-Linde et al., 2001; Marti and Bovolenta, 2002; Ruiz I Altaba et al., 2002). Moreover, Shh promotes OPCs specification from both ventral and dorsal sources (Kessaris et al., 2004). This effect of Shh is particularly important after brain injury, since it might contribute to remyelination (Amankulor et al., 2009; Ferent et al., 2013). Shh exerts its function through spatiotemporal interaction with a variety of transcription factors such as Pax6, Dlx2, Nkx2.1, Nkx2.2, Olig1, and Olig2 (Nery et al., 2001; Fuccillo et al., 2006; Xu et al., 2010; Balaskas et al., 2012). A dynamic combination of these transcription factors plays an important role in OLs differentiation (Nery et al., 2001; Nicolay et al., 2007).

Olig 1 and 2 (Oligodendrocyte Lineage Genes) are basic helix-loop-helix (bHLH) transcription factors expressed in response to ventrally secreted Shh in rodents. Gain-and-loss-of-function studies confirmed that Olig genes are necessary and sufficient for generation of OLs (Lu et al., 2000; Zhou et al., 2000; Tekki-Kessaris et al., 2001). Since the human brain is much larger, has a prolonged time of differentiation and myelination, and numerous evolutionary adaptations, the findings on OL lineage cells and Olig genes obtained in animal models cannot be directly extrapolated to the human brain. Olig genes have a great clinical relevance since they might be involved in demyelination/remyelination pathologies in MS patients and in brain lesions (Arnett et al., 2004; Fancy et al., 2004; Meijer et al., 2012). Moreover, Olig2 is selectively up-regulated after brain injury (Buffo et al., 2005), in diffuse gliomas (Ligon et al., 2004), and is linked to schizophrenia and Alzheimer's disease (Georgieva et al., 2006). Furthermore, this transcription factor has an unusual dual role in development; it first promotes expansion of the neural progenitor pool, and later affects specification and differentiation of OL lineage (reviewed in Meijer et al., 2012). Olig2 has been reported by us and others to be more involved in OL specification than Olig1 (Jakovcevski and Zecevic, 2005; Meijer et al., 2012; Mei et al., 2013). This is why in this study we focused on Olig2 progenitors, and on the effect of Shh signaling on their proliferation and differentiation in human fetal telencephalon at midgestation (ages ranging from 14 to 19 gestational weeks, gw).

We demonstrate that Shh signaling promotes Olig2^+^ cell proliferation and influences specification of RGCs into early OPCs (PDGFRα^+^), but its role in further differentiation into late OPCs is still not clear.

## Materials and methods

### Human fetal brain tissue and cell cultures

Tissues for cell cultures were dissected from human fetal forebrains (*n* = 8, Table 1) ranging in age from 14 to 19 gw, obtained from Advanced Bioscience Resources (ABR, Alameda, CA) and StemEx (Diamond Springs, CA) with proper parental consent and the approval of the Ethics Committees. No evidence of disease or abnormalities was observed after ultrasound and neuropathological examination of fetal brains. The stage of development was estimated as gw after conception and ultrasound findings. Brain tissue was collected in oxygenized Hank's balanced salt solution (Invitrogen, Carlsbad, CA) and transported on ice. Tissue from the cerebral cortex and ganglionic eminence was dissected out and dissociated mechanically and enzymatically (0.025% trypsin-EDTA, Invitrogen, Carlsbad, CA; DNAse I, 2 mg/ml, Sigma, Saint Louis, MO). Cells were seeded in expansion medium: DMEM/F12 Medium (Invitrogen, Carlsbad, CA) supplemented with B27 conditional medium (Invitrogen, Carlsbad, CA), basic fibroblast growth factor (bFGF) (10 ng/ml, Peprotech, Rocky Hill, NJ), epidermal growth factor (EGF) (10 ng/ml, Millipore, Billerica, MA) and Penicillin/Streptomycin antibiotics (Invitrogen, Carlsbad, CA) on Poly-D-Lysine (Sigma, Saint Louis, MO) coated flasks. RGC were isolated using MACS immunomagnetic sorting protocol with CD15 microbeads (Miltenyi Biotech, Auburn, CA). To isolate PDGFRα^+^ OPCs, cells were first immunolabeled with mouse anti-human CD140a antibody (1:25, BD Pharmingen, San Jose, CA) and then magnetically immunosorted by rat anti-mouse IgG2a+b secondary antibody conjugated with magnetic beads (Miltenyi Biotech, Auburn, CA).

**Table 1:** **Fetal human brain tissue used in this study**.

**Case**	**Gestational week (gw)**	**Gender**	**Direct tissue application**	**Cell culture application**
1	14	NP	–	ICC, RT-PCR, WB
2	15	♀	IHC	–
3	16	♂	RT-PCR	–
4	17	♂	–	ICC, RT-PCR, WB
5	17	♂	–	RT-PCR
6	17	NP	–	ICC
7	18	NP	RT-PCR	ICC, RT-PCR
8	19	♂	–	ICC

♀, female; ♂, male; ICC, immunocytochemistry; IHC, immunohistochemistry; NP, not provided; RT-PCR, real-time PCR; WB, western blot.

For pharmacological treatments, 2 × 10^6^ and 25 × 10^4^ cells were seeded in Poly-D-lysine coated 6-well and 24-well plates respectively. Cells were cultured for 1–3 days in expansion medium and then treated with Shh (200 ng/ml, R&D systems, Minneapolis, MN) and/or cyclopamine (2.5 μM, EnzoLife Sciences, NY) in differentiation medium (expansion medium without bFGF and EGF) applied to cells every 3 days for 14 days *in vitro* (DIV).

Three distinct differentiation media were used to promote OLs generation and maturation from RGC and OPC cultures. Apart from control medium (DMEM/F12, B27), we used OPCs expansion medium to potentiate differentiation of OPCs. This medium contained DMEM/F12, N2 conditional medium (Invitrogen, Carlsbad, CA) and PDGFaa (10 ng/ml, Peprotech, Rocky Hill, NJ). In order to get more mature OLs, we used a OLs differentiation medium and a protocol where cells were cultured first for 2 days in DMEM/F12, N2 and PDGFaa and then for an additional 14 DIV in DMEM/F12, N2, NT3 (10 ng/ml, Peprotech, Rocky Hill, NJ) and T3 (30 ng/ml, Sigma, Saint Louis, MO) (Dugas et al., 2006).

For immunohistochemistry, an additional 10 specimens were available, ranging in age from 5 gw to newborn. These cases were subject of previous reports (Jakovcevski and Zecevic, 2005; Zecevic et al., 2005; Jakovcevski et al., 2009).

### Real-time PCR

A Real-time PCR was used to determine the expression of GAPDH (Glyceraldehyde 3-phosphate dehydrogenase), Olig2, and PDGFRα. Total RNA was extracted from cells using TRIZOL^®^ reagent (Invitrogen, Carlsbad, CA) according to the manufacturer's instructions. Approximately 1 μg of RNA was used in the reverse transcription reaction using M-MuLV reverse transcriptase with random hexamers (Fermentas, Vilnus, Lithuania) according to the manufacturer's instructions. Real-time PCR was performed in a Realplex2 Mastercycler (Eppendorf, Hamburg, Germany) using 96-well reaction plates (Eppendorf, Hamburg, Germany). The reactions were prepared according to the standard protocol for one-step QuantiTect SYBR Green RT-PCR (Applied Biosystems, Cheshire, UK). The sequences 5′ → 3′ of the forward (F) and reverse (R) primers were as follows:

GAPDH:(F)ACCACCATGGAGAAGGC/(R)GGCATGGACTG TGGTCATGA
Olig2:(F)AGTCATCCTCGTCCAGCACC/(R)TCCATGGCGAT GTTGAGGT
PDGFRα:(F)AAATCTATGTTAGACTCAGAAGTC/(R) AGTAGAATCCACCATCATGCC

The thermal cycle conditions were 95°C for 2 min followed by 40 cycles of 15 s at 95°C, 15 s at 55°C and 20 s at 68°C. All assays were performed in triplicates. Averaged cycle of threshold (Ct) values of GAPDH triplicates were subtracted from Ct values of target genes to obtain ΔCt, and then relative gene expression was determined as 2−ΔCt. The results were presented relative to the control value, which was arbitrarily set to 1.

### Western blot analysis

Cells were homogenized in lysis buffer [50 mM Tris-HCl pH 7.4, 150 mM NaCl, 1% NP-40, 1 mM phenylmethylsulphonyl fluoride, and protease inhibitor cocktail] on ice for 30 min, centrifuged at 14,000 g for 15 min at 4°C, and the supernatants were collected as the cell lysates. Protein extracts obtained from cultures were separated by SDS-PAGE and electro-transferred to a nitrocellulose membrane (Bio-Rad, Hercules, CA). Membranes were blocked and incubated first with primary antibodies anti-Olig2 (1:500, Millipore) and anti-GAPDH (1:3000, Millipore) overnight at 4°C, and then with their corresponding secondary HRP-conjugated antibodies (1:15000, Thermo Fisher Scientific, Temecula, CA). Protein signal was detected using SuperSignal West Pico Chemiluminescent system (Thermo Fisher Scientific, Temecula, CA).

### Immunocytochemistry and image analysis

Cell cultures were fixed with 4% paraformaldehyde/0.2% glutaraldehyde, blocked 1 h in PBS with 0.2% bovine serum albumin (BSA) and subsequently incubated with primary antibodies diluted in phosphate buffer solution (PBS) with 0.5% BSA, 5% normal goat serum (NGS) and 0.5% Tween 20^®^ at 4°C overnight. Primary antibodies against the following proteins were used: rabbit anti-brain lipid binding protein (BLBP; 1:1000, Abcam, UK), mouse anti-Ki67 (1:50, Dako, Denmark), mouse anti-MAP2 (1:200, Sigma, Saint Louis, MO), mouse anti-NG2 (1:100, Chemicon), mouse anti-PDGFRα (1:25, Pharmingen, San Diego, CA), rabbit anti-Olig2 (1:500, Millipore), mouse anti-O4 (1:50, generous gift from Dr. Bansal), mouse anti-vimentin (1:100, Sigma, Saint Louis, MO). Primary antibodies were followed by the appropriate secondary antibodies conjugated with Alexa488 or Alexa555 fluorophores (1:500, Molecular Probes, Eugene, Oregon) for 1 h and a short incubation in bisbenzimide (Sigma) for nuclear staining.

Immunohistochemistry on frontally cut cryosections (15 μm) of fetal brains was performed as previously described (Jakovcevski and Zecevic, 2005). Tissue sections and cell cultures were visualized with the Axioskop microscope (Zeiss, Germany) using Axiovision software and photographed with a digital camera. Images were assembled in Adobe Photoshop (v. 7.0), with adjustments for contrast, brightness and color balance to obtain optimum visual reproduction of data. Immunolabeled cells from nine predesigned adjacent optical fields were analyzed by Image J software (National Institutes of Health, Bethesda, Maryland). T-test was used to discriminate between the means, with significance levels set at *p* < 0.05 and *p* < 0.01.

## Results and discussion

### Olig2^+^ cells in human telencephalon at midgestation

**Figure 1:**
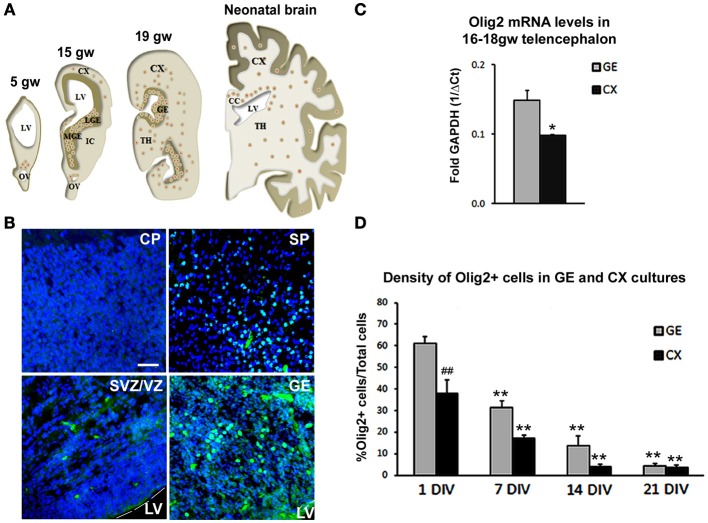
**Olig2 expression in the human fetal forebrain. (A)** Schematic representation of the distribution of Olig2^+^ cells (red dots) in frontal sections of the fetal (5–19 gw) and neonatal human telencephalon. **(B)** Representative Olig2 (green) immunostaining in 15 gw fetal forebrain. Nuclear staining with bisbenzimide (BB) in blue. **(C)** RT-PCR levels for mRNA Olig2 in CX and GE in 16–18 gw human brains. Data are presented as mean ± standard deviation. **(D)** Percentage of Olig2^+^ cells in ventral (GE) and cortical (CX) cultures. Data are presented as mean ± s.e.m. (9 optical fields quantified in each culture). ^*^p < 0.05, ^**^p < 0.01 vs. control; ^##^*p* < 0.01- GE vs. CX. Scale bar: 20 μm. CC, Corpus Callosum; CP, Cortical Plate; IC, Internal Capsule; IZ, Intermediate Zone; LGE, Lateral Ganglionic Eminence; LV, Lateral Ventricle; MGE, Medial Ganglionic Eminence; OV, Optical Vesicle; SP, Subplate layer; TH, Thalamus; VZ/SVZ, Ventricular/Subventricular Zone.

We first assessed distribution of Olig2 immunolabeled cells through increasing developmental stages, from 5–19 gw and in neonatal forebrain (Figure 1A). A considerable difference in the number of Olig2^+^ cells was observed throughout the forebrain, with higher density seen in the ventral telencephalon, in the GE, whereas in the cerebral cortex, Olig2^+^ cells were observed mainly in the subplate layer and in the ventricular/subventricular zone (VZ/SVZ), but not in the cortical plate. Example of Olig2^+^ cells distribution is shown in the 15 gw forebrain (Figure 1B). Real-time PCR analysis from human fetal brain tissues (16 and 18 gw) supported these findings and demonstrated that the levels of Olig2 mRNA were significantly higher in the GE compared to the cortex (CX) (Figure 1C). Taken together, these results are in accord with the finding that during midgestation, human OPCs are more numerous in the GE than in the cortex, suggesting their initial generation in the ventral telencephalon (Rakic and Zecevic, 2003; Jakovcevski and Zecevic, 2005; Jakovcevski et al., 2009). Scattered Olig2^+^ cells observed in the subplate layer probably represent the first cortical OPCs, as both O4^+^ and PDGFRα^+^ cells have been described in the same region and at the same developmental stages (Rakic and Zecevic, 2003; Jakovcevski et al., 2009). It is possible that the transient subplate layer, which hosts numerous afferent and efferent fibers (McConnell et al., 1989; Kostovic and Rakic, 1990; Jacobs et al., 2007), supports OPCs differentiation (Jakovcevski et al., 2009). As cortical development proceeds, enriched Olig2 expression is observed in the expanded human outer SVZ (Back et al., 2001; Rakic and Zecevic, 2003; Jakovcevski and Zecevic, 2005; Jakovcevski et al., 2009). In rodents, similar expression of Olig2 is reported in the intermediate progenitors of the SVZ and along white matter tracts, such as the corpus callosum, during perinatal development and even in the gray matter where it remains in quiescent progenitors for mature OL turnover (Levison and Goldman, 1993; Nait-Oumesmar et al., 1999; Ligon et al., 2006; Menn et al., 2006).

We were interested whether Shh regulates the expression of Olig2 during human OLs development. For that purpose, we established mixed cell cultures of dorsal (CX) and ventral (GE) regions of 17 gw forebrain. As expected from the results of immunostained cryosections, the density of Olig2^+^ cells was significantly higher in the ventral cultures compared to the dorsal (Figure 1D). Olig2^+^ cells represented 61% of total cells in ventral cultures compared to 38% in dorsal cultures 24 h post-isolation (Figure 1D). The maintenance of Olig2 in cultured cells is in line with Olig2 expression reported in the majority of progenitor cells kept in defined medium with bFGF and EGF (Hack et al., 2004). As it was observed with other neural progenitors, the number of Olig2^+^ cells decreased progressively with the withdrawal of these growth factors, which led to cell differentiation (Gabay et al., 2003; Mo et al., 2007). At 21 days *in vitro* (DIV), density of Olig2^+^ cells was only 4% in both ventral and dorsal cell cultures (Figure 1D), which is a 15 and 10 times reduction respectively from their initial numbers.

Based on the observed pattern of expression and developmental changes of Olig2^+^ cells in tissue and *in vitro*, we concluded that dissociated human cell cultures represent a reliable model for the study of human OLs development.

### Neural cell types that express Olig2 in human cortical cell cultures

**Figure 2:**
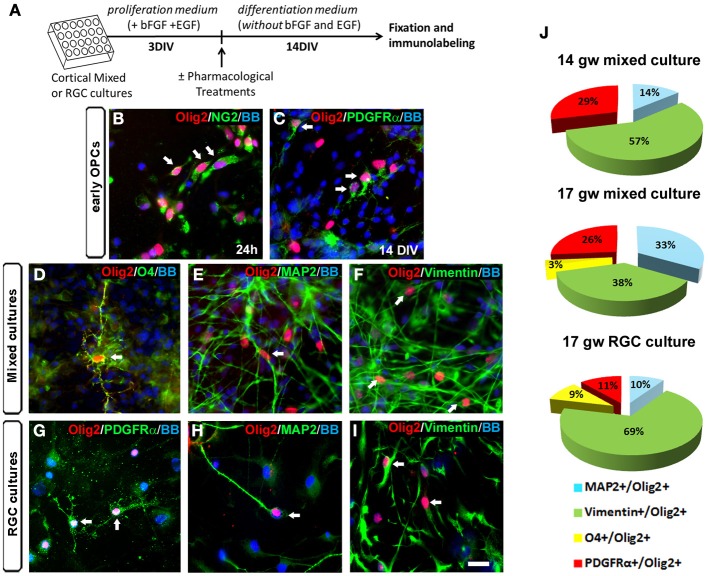
**Olig2 expression in human mixed and radial glia cell (RGC) cultures. (A)** Time-line of the cell cultures protocol used. **(B–I)** Olig2 (red) expression in mixed cell cultures in NG2^+^ cells at 24 h **(B)** and in PDGFRα^+^ early OPCs at 14 DIV **(C)**. Olig2 expression in mixed cell cultures at 14 DIV was observed in O4^+^ late OPCs **(D)**, in MAP2^+^ neurons (**E**) and in vimentin^+^ RGCs (**F**). Similar double-labeling results were demonstrated in RGC cultures after 14 DIV differentiation **(G–I)**. Arrows show double-stained cells. **(J)** The percentages of neuronal (MAP2^+^), RGC (vimentin^+^) and OPCs (PDGFRα^+^, O4^+^) cells from total Olig2^+^ cells in control mixed cell cultures (14 and 17 gw), and in 17 gw RGC culture after 14 DIV. Scale bar: 20 μm.

In rodents, even though Olig2 is mainly expressed in OLs progenitors, its presence has also been reported in astrocyte precursors in the SVZ at early postnatal stages and in reactive astrocytes after injury (Marshall and Goldman, 2002; Cahoy et al., 2008). Moreover, several studies pointed out the importance of Olig2 in the generation of neurons in the ventral telencephalon (Petryniak et al., 2007) and in the spinal cord (Lu et al., 2002; Zhou and Anderson, 2002). Indeed, progress in genetic mapping studies in rodents indicates that during CNS development, the origin of oligodendroglial lineage is more closely associated to the neurons than to astrocytes (reviewed by Meijer et al., 2012). We reported previously that Olig2 was expressed not only in human fetal OPCs but also in MAP2^+^ neurons (Jakovcevski and Zecevic, 2005). In the present study, we explored which cell types expressed Olig2 in the human fetal cortical cultures at midgestation. The expression of Olig2 was examined in cortical mixed cell cultures at 17 gw according to the experimental design presented in Figure 2A. One day after seeding cortical cells, Olig2 was observed in early OPCs labeled with NG2 (Figure 2B). After 14 DIV Olig2 was present in early PDGFRα^+^ OPCs (Figure 2C), in late OPCs marked with O4^+^ (Figure 2D), MAP2^+^ neurons (Figure 2E) and vimentin^+^ RGCs (Figure 2F). The same pattern of Olig2 distribution was observed in enriched RGC cultures from the same case (17 gw) (Figures 2G–I).

We next analyzed differentiation of Olig2^+^ progenitors in cultures from two fetal brains, aged 14 and 17 gw. At 14 gw, in mixed cortical cultures after 14 DIV, 57% of Olig2^+^ cells were co-labeled with RGC marker vimentin, 14% with neuronal marker MAP2^+^, 29% with early OPCs marker PDGFRα^+^ and no cells were labeled with late OPC marker O4 (Figure 2J). In slightly older case (17 gw), the percentage of vimentin^+^/Olig2^+^ cells was reduced to 38%; 33% were MAP2^+^, 26% were PDGFRα^+^ and 3% were O4^+^ (Figure 2J). These results, although obtained on a small number of cases, indicate that with increased fetal age from 14 to 17 gw, there is a decrease in vimentin^+^ and an increase in density of MAP2^+^ cells within Olig2^+^ population of cells. Late O4^+^ OPCs although still sparse, were first demonstrated in 17 gw mixed cultures. Obtained *in vitro* results suggest that there is a gradual differentiation of Olig2^+^ cells from multipotent RGC progenitors to neurons (19% more at 17 than at 14 gw cultures) and from early OPCs to late OPCs. Age-dependent potential of O4^+^ pre-OLs production has also been recently reported by (Cui et al., 2012). These authors observed that sorted OPCs from older fetal tissue have increased myelination potential, which argues for the existence of intrinsic mechanisms controlling OLs differentiation and myelination (Cui et al., 2012). On the other hand, comparison between mixed cultures and RGC cultures at 17 gw showed that the sum of Olig2^+^ early and late OPCs was reduced in RGC cultures (11% of PDGFRα^+^ and 9% of O4^+^). As expected, the percentage of Olig2^+^/RGCs was higher in RGC cultures (69%) compared to the mixed cultures (38%) (Figure 2J). In contrast, the percentage of MAP2^+^ neurons within Olig2^+^ population of cells was higher in mixed cell cultures (33%) compared to the RGC cultures (10%). These differences highlight the importance of distinct cellular support needed for OLs specification and differentiation that is present in mixed cell cultures and absent in enriched RGC culture. Indeed, the requirement of neurons and astrocytes to preserve OPCs proliferation and avoid their rapid loss in culture, as well as promote subsequent differentiation has been previously shown in multiple studies (Crang and Blakemore, 1997; Zhang et al., 2000; Filipovic and Zecevic, 2008; Emery, 2010; Monaco et al., 2012).

### Effect of shh on Olig2 expression

OLs specification and differentiation can be modulated by intrinsic factors, such as Olig2, but also by extrinsic cues. One of these extrinsic factors is Shh, a potent morphogen secreted early during development by the notochord and floor plate and later on by progenitor cells in ventral (Pringle et al., 1996; Orentas et al., 1999) and dorsal telencephalic regions both in rodents (Gulacsi and Lillien, 2003; Komada et al., 2008) and in humans (Mo and Zecevic, 2009). Modulation of transcription factors expression, such as Olig1 and Olig2, by Shh is both necessary and sufficient to regulate initial oligodendrogenesis at the ventral telencephalon (Lu et al., 2000; Nery et al., 2001; Cai et al., 2005; Ligon et al., 2006; Petryniak et al., 2007). Later in development, OLs are generated in dorsal cortical regions in a process often considered to be Shh-independent (Nery et al., 2001; Cai et al., 2005; Vallstedt et al., 2005; Richardson et al., 2006). However, several studies in mice reported that Shh receptors, Patched1 and Smoothened, are also expressed in dorsal cortical progenitor cells, and respond to Shh treatment by adopting OLs fate (Nery et al., 2001; Murray et al., 2002). Moreover, loss of function models of Shh and Olig1 and 2 demonstrated the importance of these extrinsic and intrinsic factors in regulation of ventrally and dorsally OLs derived populations, both in spinal cord and in forebrain (Lu et al., 2000; Kessaris et al., 2001, 2006; Fuccillo et al., 2006)

In contrast to the numerous reports on animal models, the effect of Shh signaling on OLs genesis in the human fetal brain remains elusive. Expression of Shh has been reported in human embryonic ventral spinal cord and mesencephalon (Hajihosseini et al., 1996; Orentas et al., 1999), and Shh mutation is related to brain defects such as holoprosencephaly or cyclopia (Schell-Apacik et al., 2003). Experimentally, these defects can be induced by the selective Shh inhibitor cyclopamine, a steroidal alkaloid known to interrupt Shh signaling by binding to its co-receptor Smoothened (Incardona et al., 1998). Previously, we reported that RGCs, isolated from human fetal forebrain at mid-term (20 gw), contain Shh and its receptors, Patched1 and Smoothened (Mo and Zecevic, 2009). Additionally, our unpublished results also demonstrate elements of a Gli signaling pathway in the human fetal cortical cultures (Radonjić et al., under revision). Notably, the capability of human RGC *in vitro* to generate cells of OL lineage can be modulated by Shh treatment (Mo and Zecevic, 2009). It would be particularly important to better understand the effect of Shh on dorsal oligodendrogenesis which probably plays a more significant role in the large human forebrain than in much smaller rodent brains.

**Figure 3:**
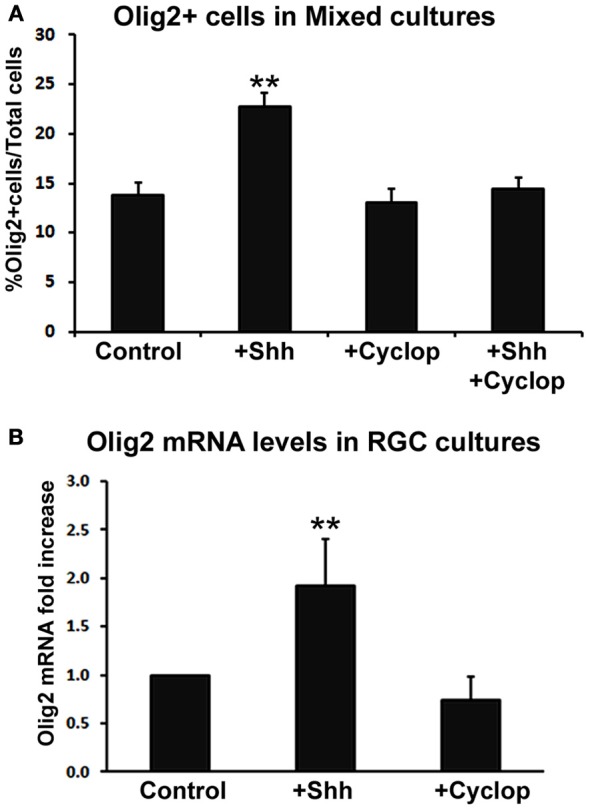
**The effect of Shh signaling on Olig2 expression *in vitro*. (A)** Treatment with exogenous Shh significantly increased the percentage of Olig2^+^ cells from total cells in human cortical mixed cell cultures, whereas either cyclopamine or cyclopamine/Shh treatment did not show any effect (*n* = 4; 14, 17, 18, and 19 gw mixed cultures). Data are presented as mean ± s.e.m. ^**^p < 0.01 vs. control. **(B)** Shh signaling modulated Olig2 mRNA levels in RGC cultures (*n* = 4; 14, two cases of 17 and 18 gw); cyclopamine treatment did not affect Olig2 mRNA levels. Data are presented as mean ± standard deviation. ^**^p < 0.01 vs. control.

We studied the effect of Shh treatment on Olig2 expression in mixed dissociated cultures obtained from human fetal cerebral cortex in four fetal brains (14, 17, 18, and 19 gw) (Figure 3A). Although we observed a trend of Shh-induced increase in the number of Olig2^+^ cells with progressively older cases, these values did not reach significance level. We, thus, combined four specimens together and compared them with controls (non Shh treated cultures). Treatment of cortical mixed cultures with Shh resulted in a significant increase in the number of Olig2^+^ cells, whereas treatment with Shh inhibitor, cyclopamine, did not affect the percentage of Olig2^+^ cells (Figure 3A). In order to demonstrate that the effect of Shh on expression of Olig2 is specific, we also treated mixed cultures with both Shh and cyclopamine. This combined treatment did not change the number of Olig2^+^ cells compared to the control, and it inhibited the increase of Olig2^+^ cells produced by the treatment with Shh alone (Figure 3A).

Similar results were observed in RGC cultures by Real-time PCR (*n* = 4; 14, 17, 17, and 18 gw). Levels of Olig2 mRNA were higher in Shh-treated cultures but not in cyclopamine-treated cultures compared to control (Figure 3B). The finding that blocking endogenous Shh did not produce an effect, suggests an additional Shh-independent regulation of Olig2 expression *in vitro*. This is consistent with a previous report on different *in vitro* and *in vivo* requirements for Shh in oligodendrogenesis (Nery et al., 2001). A possible explanation is that *in vivo* Shh is necessary to overcome a constitutive inhibition for oligodendrogenesis that does not exist *in vitro* (Nery et al., 2001; Jakovcevski et al., 2009). Besides Shh, other factors such as bFGF (basic fibroblast growth factor), are implicated in OLs development (Kessaris et al., 2004; Furusho et al., 2011). bFGF and its receptor FGFR1 are constitutively expressed in astroglia and neurons in the cortex (Leadbeater et al., 2006) and bFGF acts synergistically with Shh inducing the expression of Olig2 and the generation of OLs from cortical progenitors *in vitro* (Kessaris et al., 2004). Hence, the difference in response to pharmacological treatments observed here may be due to the different endogenous expression levels of these molecules depending on the age and the brain region studied.

### Shh promotes the generation of OPC from RGC and preserves them in undifferentiated state

**Figure 4:**
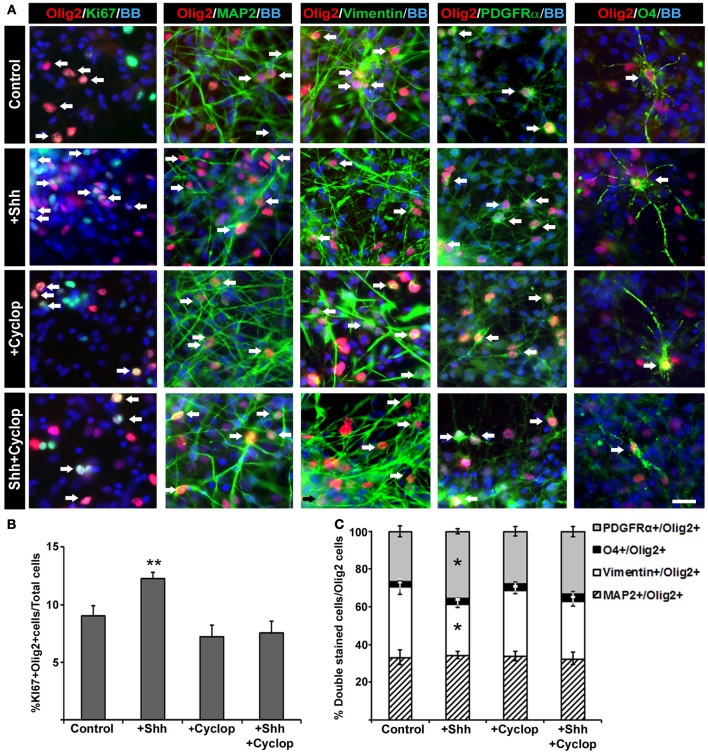
**The effect of Shh signaling on proliferation and differentiation of Olig2^+^ cells *in vitro*. (A)** In mixed cell cultures at midgestation, Olig2^+^ cells (red) were co-labeled with Ki67 (cell proliferation marker), MAP2 (neuronal marker), vimentin (RGC marker), PDGFRα(early OPC marker) and O4 (late OPC marker) in green. Nuclear staining with bisbenzimide (BB) in blue. Scale bar: 20 μm. **(B)** Proliferation of Olig2^+^ cells, estimated by double-labeling with Ki67, increased after Shh treatment, but did not change after either cyclopamine or combined cyclopamine and Shh treatments. **(C)** The composition of Olig2^+^ cell population in 17 gw cortical mixed cell cultures changed with manipulation of Shh signaling. Graph shows the percentage of Olig2^+^ neurons (Olig2^+^/MAP2^+^), RGCs (Olig2^+^/Vimentin^+^) or early (Olig2^+^/PDGFRα^+^) and late (Olig2^+^/O4^+^) OPCs out of the total number of Olig2^+^ cells; note the selective increase of Olig2/PDGFRα^+^ cells and the proportional decrease of Olig2/vimentin^+^ cells after Shh treatment. Data are presented as mean ± s.e.m. ^*^p < 0.05, ^**^p < 0.01 vs. control.

Animal studies showed that Shh increases the number of Olig2 progenitors probably by its well established function as a promoter of progenitor proliferation (Rowitch et al., 1999; Amankulor et al., 2009; Ferent et al., 2013). We analyzed the effect of Shh treatment on proliferation of Olig2 progenitors in mixed cortical cultures using proliferation marker Ki67 (Figure 4A). After 14 DIV, Shh treatment (Figure 2A) increased the number of cortical proliferative Olig2^+^ cells (Figure 4B). In mixed cultures treated with cyclopamine, or with combined Shh and cyclopamine, there was no change in the number of proliferating Olig2^+^ cells compared to the control (Figure 4B).

Next, we assessed the effect of Shh on early specification of cortical Olig2^+^ progenitors analyzing the percentages of neurons (Olig2^+^/MAP2^+^), RGCs (Olig2^+^/vimentin^+^), early OPCs (Olig2^+^/PDGFRα^+^) and late OPCs (Olig2^+^/O4^+^) within the Olig2^+^ cell population (normalized to 100% in the chart, Figure 4C). We observed that exogenous Shh induced an increase of the number of early OPCs (Olig2^+^/PDGFRα^+^) by 9% (control, 26% ± 3; Shh, 35% ± 2) at the expense of RGC (Olig2^+^/vimentin^+^) which decreased by 10% (control, 38% ± 4; Shh, 28% ± 2) (Figure 4C). Treatment with cyclopamine or combined cyclopamine and Shh did not show significant difference in the number of Olig2^+^/ PDGFRα^+^ cells and Olig2^+^/vimentin^+^ compared to control. The percentages of Olig2 expression in either MAP2^+^ neurons or O4^+^ late OPCs did not change among the treatments in 17 gw cortical mixed cultures, indicating that Shh might specifically target early OPCs (Figure 4C).

Comparable results were obtained by Real-Time PCR analysis from 14, 17, and 18 gw RGC cultures. Parallel to the increase in Olig2 mRNA levels (Control, 1 ± 0; Shh, 1.7 ± 0.3; Fold increase mean ± standard deviation), we observed that Shh treatment also enhances mRNA levels of PDGFRα (Control, 1 ± 0; Shh, 1.3 ± 0.01). On the contrary, cyclopamine treatment resulted in a significant decrease in mRNA levels of PDGFRα (0.5 ± 0.2) as well as in the mRNA levels of Olig2 (0.7 ± 0.3) in 2 of the 3 cases analyzed.

Thus, we showed here that Shh induced an increase in the number of proliferating Olig2^+^ cells as well as an increase in the total number of PDGFRα^+^/Olig2^+^ early OPCs, most likely at the expense of multipotent RG progenitors. Taking into consideration the low number of Olig2^+^/O4^+^ cells obtained in our cultures, it is difficult to assess the effect of Shh on differentiation of Olig2^+^ progenitors further into oligodendroglial lineage at the stages studied here.

### Which cells in OL lineage are the most responsive to shh?

**Figure 5:**
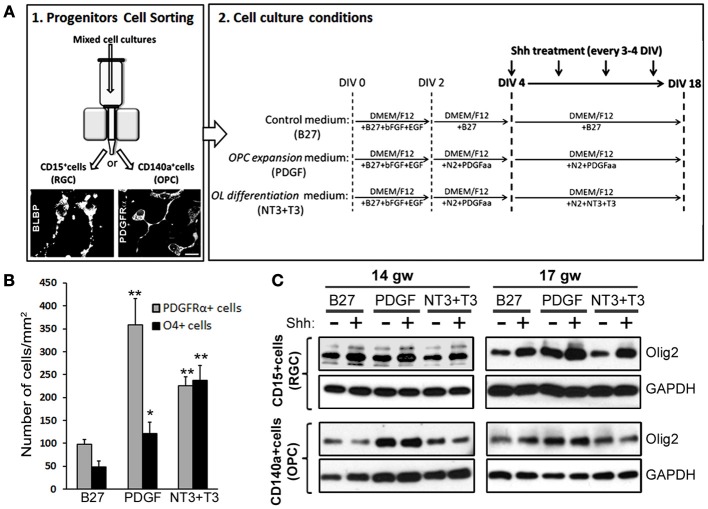
**The effect of Shh on selected cortical progenitors obtained from 14 and 17 gw cerebral cortex. (A)** Scheme of the protocol for the immunosorting of human RGCs (CD15^+^) and OPCs (CD140a^+^); Representative image of BLBP^+^ RGCs and PDGFRα^+^ early OPCs, 24 h after immunosorting. Sorted cells were cultured in three different media: Control medium (B27), PDGF and NT3+T3 media, and treated with Shh from 4-18 DIV. **(B)** Number of PDGFRα^+^ cells and O4^+^ cells per surface area in 17 gw RGCs cultures with control (B27), OPC expansion (PDGF) and OLs differentiation (NT3+T3) medium. **(C)** Protein levels of Olig2 measured by Western blot in RGCs and OPCs cultures with and without Shh treatment in the three different media. The increase levels of Olig2 protein was only observed in RGC cultures, being specially high in OPC expansion medium, and not in OPC cultures; GAPDH-loading control. Data are presented as mean ± s.e.m. ^*^p < 0.05, ^**^p < 0.01 vs. control.

To further clarify which progenitor cell type contributes the most to Shh response, we did a pilot study sorting two distinct cell populations from two fetal brains (14 and 17 gw): enriched RGC cultures (CD15 immunosorted cells) and OPC cultures (CD140a immunosorted cells). To confirm the identity of isolated cells, we performed immunostaining 24 h after isolation. For RGC that are CD15^+^, we used anti-BLBP antibody and for CD140a^+^ cells we used anti-PDGFRα antibody (Figure 5A). In order to also assess a possible differential induction of Olig2 along OL lineage, both populations of sorted cells were then cultured for 18DIV with three distinct media that promote differentiation into different maturation stages of OLs lineage (Figure 5A): (a) control medium supplemented with B27 (B27), (b) medium supplemented with N2 and PDGFaa, a potent promotor of OPC proliferation (PDGF) (Hu et al., 2012), and (c) medium initially supplemented with N2 and PDGF and afterwards with NT3 and T3 (NT3+T3) to obtain late OPCs/early OLs (Dietrich et al., 2002; Dugas et al., 2006; Neri et al., 2010; Cui et al., 2012). We tested the effect of the three different media in RGC cultures, and as expected, PDGF medium produced the highest number of PDGFRα^+^ cells (Figure 5B). In the NT3+T3 medium, where cells were exposed to PDGFaa for 2 days, we observed a significant increase in the number of PDGFRα^+^ cells compared to the control, B27 medium. The highest levels of O4^+^ cells were observed in NT3+T3 maturation medium (Figure 5B). These results confirmed the effects of PDGFaa and NT3+T3 on OPC proliferation and maturation respectively.

We next showed that in RGC cultures Olig2 protein levels increased after the Shh treatment regardless of the culture medium, while no changes were observed in OPC cultures (Figure 5C). This difference suggests that RGC progenitors are more susceptible to Shh and increase their Olig2 content in response to Shh treatment. The highest level of Olig2 after Shh treatment was demonstrated in PDGF medium (Figure 5C). Under these conditions the number of early OPCs (PDGFRα^+^) was also the highest (Figure 5B). This result indicates that Shh and PDGF might synergistically promote the differentiation of RGCs into OPCs, through the increase in the expression of Olig2. The lack of induction in OPC cultures might indicate that once cells are committed to OL fate, they do not upregulate Olig2 in response to Shh. Similar *in vitro* results were reported for mouse embryonic stem cells. Treatment with Shh induced Olig2 expression in progenitor cells until oligodendrogenesis starts, but Shh was not required for the differentiation into later stages of OL lineage (Du et al., 2006). Similarly, in the spinal cord Shh also promotes proliferation of selected CNS precursors and blocked their differentiation (Rowitch et al., 1999).

### Subcellular localization and function of Olig2 in human developing OLs

**Figure 6:**
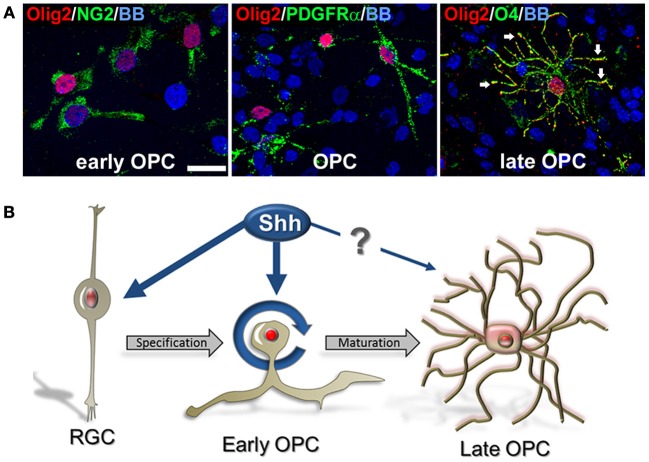
**Cellular distribution of Olig2 in different cell types. (A)** Nuclear expression in early OPCs NG2^+^ (1 DIV) and PDGFRα^+^ (14 DIV) cells and cytosolic expression in O4^+^ late OPCs/immature OLs (14 DIV) (arrows). Nuclear staining with bisbenzimide (BB) in blue. Scale bar: 20 μm. **(B)** Schematic presentation of the possible effect of Shh on Olig2 expression (red) along the OLs lineage in human fetal cortical cultures. Shh might actively participate in proliferation and specification of early OPCs from RGCs in the cerebral cortex. However, the effect of Shh and Olig2 in the differentiation of early OPCs to mature OLs might not be required.

We observed that Olig2 expression was generally nuclear in various cell types, consistent with its role as a transcription factor. However, with the progression of OLs development, Olig2 can also be observed in the soma of the cells. In late OPCs, labeled with O4 antibody, Olig2 is observed in the increasingly ramified cell processes in contrast to a restricted nuclear localization in early OPCs (Figure 6A). This finding supports our previous results on cellular localization of Olig2 in the human developing brain (Jakovcevski and Zecevic, 2005; Jakovcevski et al., 2009). The control of subcellular localization of transcription factors is considered to represent a regulatory mechanism of many signaling pathways (Ziegler and Ghosh, 2005). Thus, different subcellular localization of Olig2 at different OL maturation stages might be due to a different requirement of this transcription factor during OLs development. Recent reports on conditional knock out animals demonstrate that deletion of Olig2 in OLs progenitors (CNP^+^ cells) dramatically reduces differentiation into OLs, whereas Olig2 ablation in mature OLs (PLP^+^) enhances maturation and increases myelination (Mei et al., 2013). This key role of Olig2 in OL specification and maturation might clarify why the transplantation of Olig2-overexpressing neural stem cells into demyelinating lesions significantly enhances the generation of OL but has only modest effects on remyelination (Copray et al., 2006; Maire et al., 2009; Kim et al., 2011; Hu et al., 2012).

## Concluding remarks

Numerous developmental studies tried to clarify regulatory mechanisms of Shh and Olig2 on oligodendrogenesis in animal models, but similar studies have not been done in developing human brain. We demonstrated that Shh promotes an expansion of human cortical progenitors, similar to its effect in rodents. Shh also influences progenitor fate by enlarging the pool of Olig2^+^/PDGFRα^+^ early OPCs. Based on our results we propose that Shh might actively participate in proliferation and specification of early cortical OPCs from RGCs, but its role in the transition from early OPCs to mature OLs is still unclear (Figure 6B). Better understanding of Shh and Olig2 function in human OL development is a critical point in developing new therapeutic targets for demyelinating diseases.

### Conflict of interest statement

The authors declare that the research was conducted in the absence of any commercial or financial relationships that could be construed as a potential conflict of interest.
